# Laparoscopic versus open liver resection for colorectal liver metastasis: an umbrella review

**DOI:** 10.3389/fonc.2024.1340430

**Published:** 2024-07-15

**Authors:** Federico Pinto, Marco Di Pangrazio, Alessandro Martinino, Letizia Todeschini, Francesco Toti, Luca Cristin, Miriam Caimano, Amelia Mattia, Giuseppe Bianco, Gabriele Spoletini, Francesco Giovinazzo

**Affiliations:** ^1^ Department of Surgery, University of Illinois at Chicago, Chicago, IL, United States; ^2^ Faculty of Medicine and Surgery, University of Verona, Verona, Italy; ^3^ General Surgery and Liver Transplant Unit, Fondazione Policlinico Universitario Agostino Gemelli IRCCS, Rome, Italy; ^4^ Surgical Department, San Camillo Hospital, Treviso, Italy; ^5^ Department of Surgery, UniCamillus-Saint Camillus International University of Health Sciences, Rome, Italy; ^6^ Unit of General and Liver Transplant Surgery, Department of Medical and Surgical Sciences, Agostino Gemelli University Polyclinic (IRCCS), Rome, Italy

**Keywords:** minimally invasive surgery, laparoscopic liver resection, open liver resection, outcomes, colorectal liver metastasis

## Abstract

**Introduction:**

This study comprehensively compared laparoscopic liver resection (LLR) to open liver resection (OLR) in treating colorectal cancer liver metastasis (CRLM).

**Methods:**

A systematic review of relevant literature was conducted to assess a range of crucial surgical and oncological outcomes.

**Results:**

Findings indicate that minimally invasive surgery (MIS) did not significantly prolong the duration of surgery compared to open liver resection and notably demonstrated lower blood transfusion rates and reduced intraoperative blood loss. While some studies favored MIS for its lower complication rates, others did not establish a statistically significant difference. One study identified a lower post-operative mortality rate in the MIS group. Furthermore, MIS consistently correlated with shorter hospital stays, indicative of expedited post-operative recovery. Concerning oncological outcomes, while certain meta-analyses reported a lower rate of cancer recurrence in the MIS group, others found no significant disparity. Overall survival and disease-free survival remained comparable between the MIS and open liver resection groups.

**Conclusion:**

The analysis emphasizes the potential advantages of LLR in terms of surgical outcomes and aligns with existing literature findings in this field.

**Systematic review registration:**

[website], identifier [registration number].

## Introduction

Colorectal cancer’s tendency to spread to the liver poses a substantial treatment challenge (1). Traditional open surgical approaches, while effective, entail considerable morbidity and protracted recovery periods. The foundation of minimally invasive resection lies in applying laparoscopic and robotic-assisted techniques. Laparoscopy, introduced in the 1980s, revolutionized surgery by enabling internal organ visualization and manipulation through small incisions. This technique mitigates tissue trauma, reducing pain and quicker postoperative recovery (2). Robotic-assisted surgery further enhances precision and dexterity through robotic arms operated by surgeons (3). Recent research underscores the efficacy and safety of minimally invasive liver resections for CRLM, demonstrating comparable oncological outcomes to traditional open surgeries, reduced blood loss, diminished post-operative complication rates, and shorter hospital stays (4, 5). The less invasive nature of these procedures augments patient satisfaction and cosmesis, thus improving overall quality of life during the recovery phase.

Nonetheless, refining patient selection criteria and optimizing techniques for complex cases remain ongoing challenges. Advancements in imaging technologies, intraoperative navigation systems, and instrumentation continually shape the minimally invasive liver surgery landscape. Ongoing research endeavors are dedicated to unraveling long-term oncological outcomes and refining the technical facets of these procedures. This umbrella review explores the safety and efficacy of laparoscopic liver resection (LLR) in contrast to open liver resection (OLR) for the treatment of colorectal liver metastases (CRLM).

### Historical development

The transformative period in hepatic surgery during the early 1990s witnessed the emergence of laparoscopic liver resection (LLR). Pioneering efforts by Reich et al. (6), Katkhouda et al. (7), and Gagner et al. (8) in 1991 and 1992 inaugurated this revolutionary approach, heralding a new era in surgical techniques. Building upon this foundation, subsequent years saw substantial advancements, including the groundbreaking left lateral sectionectomy (LLS) in 1996 and the progressive evolution toward hepatectomy by 1998 (9, 10). These sequential developments underscored the swift progression of LLR methodologies, adapting to the ever-evolving landscape of surgical innovation.

The expansion of LLR procedures mirrored the historical evolution observed in open liver resections (OLR). In 2009 Nguyen et al. published the first international multicenter study supporting the idea that laparoscopic liver resection for colorectal cancer metastasis was safe, feasible, and comparable in terms of oncologic outcomes to open liver resection. The significance of LLR was underscored by two pivotal international consensus conferences held in 2008 in Louisville and 2014 in Morioka (11, 12). The first one focused on the viability of LLR, and the second conference centered around contrasting laparoscopic approaches with the then-standard open resection procedure, highlighting the evident relevance of a laparoscopic approach in the contemporary landscape of liver surgery. A third international conference took place in Seoul, Korea, in 2016. During this event, a panel of experts concentrated their efforts on formulating a statement concerning laparoscopic living donor hepatectomy (13). In 2017 the first European guidelines meeting took place in Southampton, where the primary objective was to present and validate clinical practice guidelines concerning laparoscopic liver surgery (14).

These consensus conferences provided a platform for leading experts to convene, discuss, and deliberate upon the state of LLR, sharing insights and perspectives that shaped its trajectory. Moreover, these conferences fostered a dynamic space for exchanging knowledge and best practices, facilitating the dissemination of advancements and fostering a global dialogue on LLR’s progress. One of the critical considerations that emerged on this transformative journey was the management of LLR-specific complications. This concern was effectively addressed through meticulous procedural implementation and the systematic evaluation of outcomes. As careful application and patient assessment became routine, these efforts alleviated anxieties and validated the advantages of LLR over its conventional counterpart, OLR.

## Materials and methods

In the umbrella review, a comprehensive search and analysis of various systematic reviews and meta-analyses concerning minimally invasive surgery (MIS) in liver resections for colorectal cancer (CRC) was conducted, as previously described (15). This study adhered to an already established research protocol (16). An AMSTAR 2 checklist is provided as **Supplementary Material** to assist in the evaluation and assessment of the systematic review presented herein (17).

### Objectives and PICO process

The primary objective of this umbrella review is the assessment of postoperative mortality and overall/disease-free survival in the two analyzed groups. The secondary objectives encompass assessing parameters such as blood loss, blood transfusion, duration of surgery, complication rate, hospitalization time, surgical margins R0, and recurrence.

Utilizing the PICO criteria in framing a research question, the study aimed to investigate the following: “In patients undergoing surgical treatment for CRC liver metastasis (P), does laparoscopic surgery (I) compare to traditional open surgery (C), result in differences in postoperative mortality, overall/disease-free survival, blood loss, blood transfusion requirements, duration of surgery, complication rate, hospitalization time, surgical margins, and recurrence (O)?” (18).

### Search strategy

The systematic review adhered to the guidelines outlined in the PRISMA statement for the conduct and reporting of data (19). The research encompassed an exhaustive computerized exploration of the PubMed and Cochrane Library databases. Employing an advanced search strategy, we employed terms such as “colorectal neoplasm” OR “colorectal” AND “liver metastases” AND “liver neoplasm” AND “therapeutics” OR “treatment” AND “meta-analysis” OR “systematic review”. Results were admitted from the time of inception up to and including June 7, 2023. Moreover, manual screenings of reference lists from pertinent articles were conducted, aiming to identify further relevant studies.

### Inclusion and exclusion criteria

Articles were eligible for inclusion if they were systematic reviews or meta-analyses focusing on patients with CRC and liver metastasis. The selected articles were required to analyze laparoscopic liver resections versus open liver resections performed in individuals who were 18 years of age or older. We excluded all non-English language studies.

### Data extraction

At least two reviewers independently gathered all data, resolving discrepancies through collaborative discussion and consensus. The diverse outcomes within different meta-analyses were independently extracted to ensure a meticulous and nuanced collection of data. Data collection encompassed the following information: authorship details, year of publication, the number of articles scrutinized, and the number of patients enrolled in each study. Additionally, the review calculated pooled outcome measures, presented values with 95% confidence intervals (95% CI), assessed statistical heterogeneity, and evaluated potential publication bias. Furthermore, a quality assessment of the included meta-analyses was conducted using the specific quality assessment tool developed by the Centre of Evidence-Based Medicine at the University of Oxford (**Table 1**).

**Table 1:** Quality assessment of the meta-analysis.

Study	PICO Question	Search strategy	Inclusion criteria	Heterogeneity and methods to address the heterogeneity	Quality assessment of included studies	Publication bias evaluation
Tian ZQ. et al. (20)	A meta-analysis was conducted to evaluate the benefits of laparoscopic compared with open liver resection for treatment colorectal liver metastases.	MEDLINE (PubMed), EMBASE and CENTRAL databases covering studies published until October 18th 2016 and a manual approach.	(1) Study design: comparing laparoscopic with open liver resection for colorectal liver metastases patients (2). Each group includes more than 10 patients (minimum of 20 patients) (3). The studies provided surgical and oncologic outcomes, and (4) available data for each surgical regimen.	Heterogeneity was assessed using I-squared (I 2) test and P value.	The quality assessment for each study was performed with Newcastle-Ottawa Scale (NOS).	Funnel plots were used to assess potential publication bias
Wei M. et al. (21)	Meta-analysis to compare the outcomes of laparoscopic versus open liver resections.	A systematic search was conducted in the PubMed and EmBase Databases	(1) Diagnosis of colorectal cancer liver metastasis in adult patients. (2) The surgical procedure compares laparoscopic and open approaches. (3) The studies provides short- or long-term outcomes, and (4) available data for each surgical regimen.	Heterogeneity was measured with the I2 index and P value	The Newcastle-Ottawa Scale (NOS) was used to assess selection, comparability and outcomes.	Publication bias was assessed with funnel plots.
Zhou Y. et al. (22)	A meta-analysis was conducted to assess the quality of evidence in the literature, thereby strengthening the basis for recommending laparoscopic liver resection (LLR) as a viable alternative to open liver resections (OLR) for the treatment of colorectal liver metastases (CLM).	MEDLINE, EMBASE, OVID, and Cochrane database were searched to identify all clinical trials published as full papers in the English language that compared LLR and OLR for CLM between July 1992 and March 2013.	(1) A study had to compare LLR and OLR for CLM. (2) In cases where dual or multiple studies originated from the same institution, only the most recent study was considered for analysis.	Heterogeneity was assessed using χ2 and I2 statistics. Data exhibiting non-significant heterogeneity (P > 0.1) were analyzed using a fixed-effects model, while heterogeneous data (P < 0.1) were subjected to calculations employing a random-effects model.	The Newcastle-Ottawa Scale (NOS) was used to assess the comparability of the two study groups, and assessment of outcomes.	Publication bias was evaluated through visual examination using a funnel plot, plotting standard error against the effect size (log odds ratio).
Pan L. et al. (23)	This meta-analysis was undertaken to compare laparoscopic liver resection (LLR) and open liver resection (OLR) approaches, focusing on intraoperative and postoperative complications, as well as long-term outcomes. The analysis was conducted based on the existing literature.	A systematic search of online databases, including PubMed, Web of Science, Cochrane Library, and Embase, was conducted to identify pertinent studies comparing open surgeries with laparoscopic surgeries for the simultaneous resections of colorectal cancer (CRC) and synchronous colorectal liver metastases (SCRLM) up until June 5, 2019.	(1) Patients with proven or suspected synchronous colorectal liver metastases (SCRLM), where liver metastasis was detected simultaneously with the detection of colorectal cancer (CRC). (2) Laparoscopic versus open surgeries for simultaneous resections. (3) Randomized controlled studies or observational studies, including cohort and case–control studies. (4) Studies reporting at least one outcome of either perioperative results or long-term outcomes. (5) A study population of more than 20 patients. (6) Only full-length articles were considered for inclusion.	Heterogeneities among studies were assessed using the Cochran Chi-square test and I2 index.	The Newcastle–Ottawa scale (NOS) was used to assess the quality of the included studies.	Funnel plots, Harbord tests, Peters tests, and Egger tests were used to detect any publication bias.
Luo LX. et al. (24)	This meta-analysis comprehensively assesses all available evidence, incorporating both controlled trials and observational studies. The objective is to discern the preferred surgical approach for patients with colorectal cancer liver metastases (CRCLM).	Electronic databases, including the Cochrane Central Register of Controlled Trials (CENTRAL), PubMed, and Embase, underwent a systematic search to identify all relevant studies published prior to June 2013.	(1) a comparative study that involved both laparoscopic hepatectomy (LH) and open hepatectomy (OH) groups for colorectal cancer liver metastasis (CRCLM); (2) hepatectomy without simultaneous resection of the primary colorectal cancer (CRC); (3) availability of full-text; (4) inclusion of descriptions for at least one perioperative or oncologic outcome; and (5) in cases where two or more reports shared the same or overlapping population and data, only the most recent, comprehensive, or high-quality article was considered for inclusion.	Heterogeneity was quantified by the I2 index.	The quality assessment of all incorporated observational studies was conducted using the Newcastle–Ottawa Scale (NOS).	Funnel plots and Begg rank correlation were used to assess publication bias.
Guo Y. et al. (25)	This meta-analysis was undertaken to compare the perioperative results and long-term outcomes of simultaneous resections for colorectal cancer (CRC) and synchronous colorectal liver metastases (SCRLM) between the laparoscopic and open approach.	A systematic search was conducted in the Cochrane Library, PubMed, EMBASE and Ovid databases for all the years (until May 5, 2016).\	(1) Synchronous colorectal liver metastases (SCRLM) were defined as metastases either detected at the time of the primary colorectal cancer (CRC) detection or within six months of CRC presentation. (2) The minimally invasive approach (MIA) for simultaneous CRC and SCRLM resections was compared with the open approach (OA) in the study. Minimally invasive simultaneous resections were considered if totally laparoscopic, hand-assisted, or robot-assisted techniques were employed for resections of CRC and SCRLM during a single procedure. (3) The studies reported at least one primary outcome related to perioperative results or long-term outcomes. (4) Only studies published or accepted for publication as full-length articles were included.	The I2 test was used to calculate the heterogeneity across studies.	The Newcastle-Ottawa Scale (NOS) was used to assess the quality of the studies included.	A funnel plot was employed as a tool to assist in interpreting the potential presence of publication bias.
Ye SP et al. (26)	This meta-analysis aimed to compare the short- and long-term outcomes of Minimally Invasive Surgery (MIS) and Open Surgery (OS) for the simultaneous resection of primary colorectal cancer (CRC) and synchronous colorectal liver metastases (SCRLM), drawing insights from the current available literature.	A comprehensive search was carried out on the Web of Science, Cochrane Library, Embase, and PubMed databases to locate pertinent studies The search spanned studies published until December 22, 2018.	(1) Comparative assessment of treatment outcomes between Minimally Invasive Surgery (MIS) and Open Surgery (OS) for the simultaneous resection of colorectal cancer (CRC) and synchronous colorectal liver metastases (SCRLM), with MIS limited to laparoscopic or robotic-assisted procedures; (2) Acceptance or publication of papers with available full texts; (3) Inclusion of articles reporting on a minimum of three treatment outcomes from the list provided below.	The assessment of heterogeneity among the studies was performed using the I2 statistic.	The Newcastle-Ottawa scale (NOS) was used to evaluate the methodological quality of the studies.	Publication bias was ascertained through the examination of a funnel plot and assessed using both the Begg’s test and Egger’s test.
Schiffman et al. (27)	The objective of this study was to systematically analyze clinical evidence in case-matched studies comparing laparoscopic liver resection (LLR) with open liver resection (OLR) in patients with metastatic colorectal cancer (mCRC).	Two authors independently conducted electronic literature searches using PubMed to identify studies that compared laparoscopic liver resection (LLR) with open liver resection (OLR) in patients with metastatic colorectal cancer (mCRC).	(1) Comparison of laparoscopic liver resection (LLR) with open liver resection (OLR) in patients undergoing resection for metastatic colorectal cancer (mCRC). (2) Each group comprising a minimum of 10 patients, with a requirement of at least 20 patients overall. (3) Reporting on at least one of the specified outcomes mentioned herein.	The I-squared statistic was used to assess heterogeneity among studies.	Not reported	No publication bias evaluation available
Kelly et al. (28)	The objective of this review was to systematically assess the existing evidence from matched population studies that compare open and laparoscopic liver resection for the management of colorectal liver metastases (CRLM).	A comprehensive electronic search for pertinent publications was conducted utilizing the following resources: PubMed, Embase, Ovid, and the Cochrane Collaboration database. The search encompassed the period from January 2000 to January 2020.	(1) report on patients with only colorectal liver metastasis (CRLM). (2) compare the approaches for management of CRLM (open versus laparoscopic liver resection). (3) patient populations across the studies must be matched. (4) report on surgical and outcomes measures mentioned below. (5) have a clear research methodology.	Heterogeneity was assessed by I-squared statistics	The quality assessment of the studies included in this systematic review was conducted using the Methodological Index for Non-Randomized Studies (MINORS) score.	Not reported
Ozair et al. (29)	The objective of this study was to conduct a systematic review and meta-analysis of the literature to compare the efficacy, effectiveness, and safety of minimally invasive surgery (MIS) versus open hepatectomy for resectable colorectal liver metastases (CRLM).	A comprehensive electronic search for pertinent publications was conducted utilizing the following resources: PubMed, Embase, Cochrane CENTRAL, ClinicalTrials.gov, International Clinical Trials Registry Platform (ICTRP), and Google Scholar.	(1) Peer-reviewed randomized controlled trials and non-randomized comparative studies, published in English. (2)Studies involving adult patients (aged 18 years or older) diagnosed with colorectal cancer and resectable colorectal liver metastases (CRLM) who were undergoing surgery. (3) Inclusion of all studies comparing open and minimally invasive surgery (MIS) approaches. MIS was defined to encompass laparoscopic, laparoscopic hand-assisted, robotic, and hybrid approaches.	I2 and χ2 statistics were used to assess heterogeneity.	For quality assessment of randomized controlled trials (RCTs), the Cochrane Risk of Bias (RoB) 2.0 Tool was used. For non-randomized comparative studies, a modified Newcastle-Ottawa Scale (NOS).	A funnel plot was employed as a tool to assist in interpreting the potential presence of publication bias.
Syn et al. (30)	An individual participant data (IPD) meta-analysis was conducted to address the current shortage of high-quality evidence regarding the impact of minimally-invasive surgery on longterm oncological outcomes, especially overall survival (OS). This analysis includes data from randomized trials and propensity-score matched (PSM) studies that compare laparoscopic and open hepatectomy for colorectal liver metastases (CLM).	A comprehensive search was carried out on EMBASE, Scopus and Medline (via Ovid) for randomized and propensity-score matched (PSM) studies without language restriction from inception to July 22, 2019.	(1) Randomized trials and propensity-score matched (PSM) studies comparing the effect of laparoscopic versus open resection of CLM on Overall Survival. (2) Meeting abstracts and unpublished online data were considered for inclusion if they contained Kaplan-Meier survival curves and provided sufficient information regarding treatment and patient characteristics.	Heterogeneities among studies were assessed using the Chi-square and I-square statistics.	The Newcastle-Ottawa Scale (NOS) was used to assess the quality of the non-randomized studies included. Risk of bias in randomized trials was assessed using the Cochrane Risk-of-Bias tool.	Publication bias was assessed with funnel plots.

## Results

**Figure 1:**
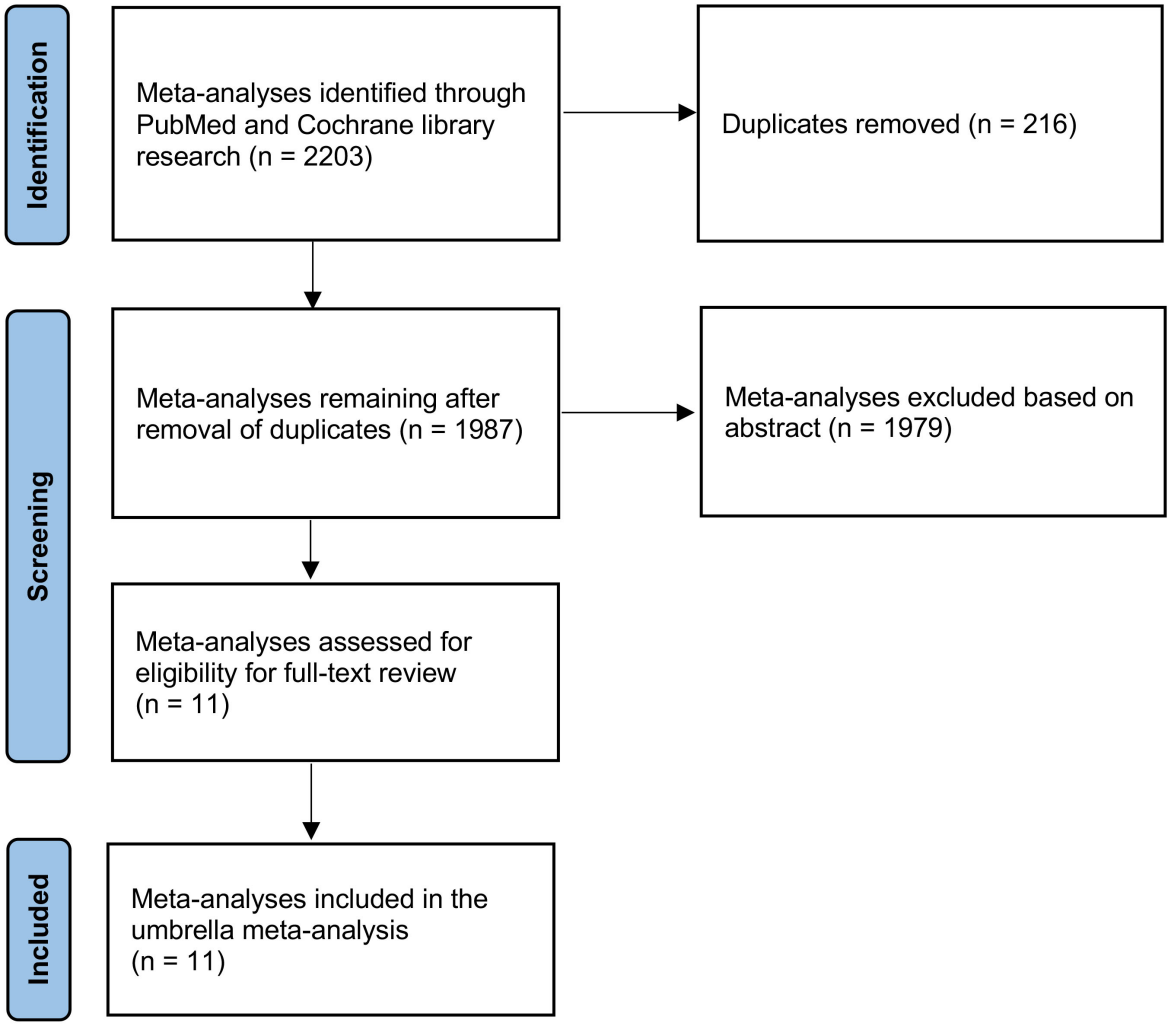
PRISMA flow diagram.

An extensive search retrieved a total of 2203 records. During the initial screening phase, 216 articles were excluded due to duplication, and 1979 were excluded for not meeting the inclusion criteria. Consequently, only eleven articles remained eligible for a thorough full-text review (**Figure 1**).

Ultimately, our study comprised eleven included articles, all of which were meta-analyses (**Table 2**) (20–30). In **Table 3**, we present the analyzed outcomes comparing minimally invasive hepatectomy with open hepatectomy for CRLM.

**Table 2:** Features of articles included.

Author et al.	Year	Type of study	Number of studies	Number of patients
Tian ZQ. et al. (20)	2016	Meta-Analysis	14	° 1679 LLR: 683, OLR: 996
Wei M. et al. (21)	2013	Meta-Analysis	14	° 975 LLR: 376, OLR: 599
Zhou Y. et al. (22)	2013	Meta-Analysis	8	° 695 LLR: 268, OLR: 427
Pan L. et al. (23)	2020	Meta-Analysis	12	° 616 LLR: 273, OLR: 343
Luo LX. et al. (24)	2014	Meta-Analysis	7	° 624 LLR: 241, OLR: 383
Guo Y. et al. (25)	2018	Meta-Analysis	6	° 377 LLR: 164, OLR: 213
Ye SP et al. (26)	2019	Meta-Analysis	10	° 502 LLR: 216, OLR: 286
Schiffman et al. (27)	2015	Meta-Analysis	8	° 610 LLR: 242, OLR:368
Kelly et al. (28)	2022	Meta-Analysis	14	° 3095 LLR: 1314, OLR: 1781
Ozair et al. (29)	2022	Meta-Analysis	35	* 506 LLR: 245, OLR: 261 ° 2294 LLR: 1017, OLR: 1567 857 LLR: 346, OLR: 511
Syn at al (30).	2020	Meta-Analysis	15	* 473 LLR: 229, OLR: 244 °2675 LLR: 1046, OLR: 1629

*Only RCT.

°Only NRCT.

Simultaneous resection of CRLM and primary CRC. LLR, laparoscopic liver resection; OLR, open liver resection.

**Table 3:** Results for different outcomes in patients undergoing laparoscopic versus open liver resection for CRLM.

Outcomes	Reference	SMD/MD/OR/ RR/HR	95% CI	P-value	Test of heterogeneity
Blood loss	(20)° (21)° (22)° (24)° (26)° (27)° (28)° (29)* (29)° (30)	-216.7 -182.87 -173.08 -188.858 -130.09 0.70 0.72 -251.61 -178.80 /	-309.4,-124.1 -263.50, -102.25 -297.52, -48.64 -294.033, -83.682 -210.95, -49.23 0.00, 1.41 0.39, 1.05 -555.45, 52.23 -234.50, -123.11 /	< 0.00001 < 0.0001 0.006 0.001 0.002 0.049 0.0001 0.10 < 0.00001 /	I2 89%, P < 0.00001 I2 90%, P < 0.00001–0.0008 I2 83% I2 39.1% I2 91%, P < 0.00001 I2 91.1%, P= 0.000 I2 91% I2 85%, P = 0.001 I2 92%, P < 0.00001 /
Blood transfusion	(20)° (21)° (22)° (24)° (26)° (27)° (28)° (29)* (29)° (30)	0.36 0.41 0.35 0.44 0.35 1.96 1.74 0.81 0.54 /	0.23, 0.55 0.24, 0.69 0.20, 0.64 0.267, 0.711 0.29, 0.95 1.24, 3.09 1.30, 2.33 0.45, 1.49 0.39, 0.75 /	0.47 0.0008 0.001 0.001 0.03 0.004 0.03 0.50 0.0002 /	I2 0%, P < 0.00001 I2 7%, P = 0.37 I2 0% I2 0%, P = 0.635 I2 0%, P = 0.81 I2 0.0%, P = 0.755 I2 33%, P ≤ 0.0002 I2 0%, P = 0.35 I2 0%, P = 0.77 /
Duration of surgery	(20)° (21)° (22)° (24)° (26)° (27)° (28)° (29)* (29)° (30)	3.01 5.10 1.91 3.05 34.05 0.05 -0.07 / / /	-11.6, 17.6 -8.92, 18.94 -15.92, 19.75 -14.394, 20.494 0.65, 67.46 -0.44, 0.54 -0.16, 0.02 / / /	0.69 0.48 0.83 0.732 0.05 0.85 0.11 / / /	I2 31%, P = 0.16 I2 60%, P = 0.004 I2 49% I2 2.4%, P = 0.401 I2 84%, P < 0.00001 I2 83.5%, P = 0.000 I2 82%, P ≤ 0.001 / / /
Complication rate	(20)° (21)° (22)° (24)° (26)° (27)° (28)° (29)* (29)° (30)	0.31 0.57 0.56 0.647 0.78 1.41 1.58 0.62 0.53 /	0.47, 0.80 0.42, 0.78 0.39, 0.82 0.477, 0.877 0.51, 1.18 1.04, 1.92 1.19, 2.09 0.38, 1.00 0.38, 0.74 /	0.003 0.0005 0.003 0.005 0.24 0.03 0.002 0.05 0.0002 /	I2 95% / I2 0% I2 0% I2 0%, P = 0.89 I2 0.0%, P = 0.484 I2 0%, P = 0.45 I2 0%, P = 0.54 I2 0%, P = 0.84 /
Hospitalization Time	(20)° (21)° (22)° (24)° (26)° (27)° (28)° (29)* (29)° (30)	-3.85 -3.39 -3.54 -2.641 -4.06 1.50 / -6.61 -2.67 /	-5.00, -2.71 -4.29, -2.48 -5.12, -1.96 -5.588, 0.306 -5.95, -2.18 0.41, 2.60 / -10.19, -3.03 -3.27, -2.07 /	< 0.00001 < 0.00001 < 0.001 0.079 < 0.0001 0.007 0.001 0.0003 0.00001 /	I2 70%, P < 0.0001 66%, P <0.00001 I2 75% I2 86.95%, P < 0.01 I2 69%, P = 0.006 I2 96.7%, P = 0.000 / I2 0%, P = 0.45 I2 53%, P = 0.004 /
Post-operative mortality	(20)° (21)° (22)° (24)° (26)° (27)° (28)° (29)* (29)° (30)	0.48 -0.01 0.69 0.625 / / / / / /	0.15, 1.57 -0.03, 0.01 0.13, 3.75 0.12, 3.25 / / / / / /	0.23 0.58 0.67 0.576 / 0.92 / / / /	I2 0%, P = 0.95 I2 0%, P = 1.00 I2 0% I2 0% / / / / / /
Surgical Margins R0	(20)° (21)° (22)° (24)° (incidence of R1) (26)° (27)° (incidence of R1) (28)° (29)* (29)° (30)	1.50 2.44 2.97 0.357 / / 0.72 1.08 1.01 /	1.03, 2.18 1.21, 4.94 1.53, 5.78 0.180, 0.708 / / 0.57, 0.90 1.00, 1.17 0.99, 1.02 /	0.04 0.01 0.001 0.003 / 0.36 0.005 0.06 0.54 /	I2 25%, P = 0.21 / I2 0% I2 0% / / I2 11%, P = 0.34 / I2 3%, P = 0.42 /
Recurrence	(20)° (21)° (22)° (24)° (26)° (27)° (28)° (29)* (29)° (30)	0.78 0.70 0.68 / / / / / / /	0.61, 0.99 0.44, 1.12 0.41, 1.14 / / / / / / /	0.04 0.14 0.14 / / / / / / /	I2 18%, P = 0.29 I2 0%, P = 0.57 I2 0% / / / / / / /
Disease Free Survival	(20)° (5 year) (21)° (22)° (5 year) (24)° (26)° (27)° (28)° (29)* (1 year) (29)* (3 year) (29)* (5 year) (29)° (1 year) (29)° (3 year) (29)° (5 year) (30)	0.88 / 1.48 1.234 1.00 / / 1.03 1.08 1.02 1.05 1.04 1.10 /	0.53, 1.47 / 0.89, 2.44 0.652, 2.333 0.67, 1.50 / / 0.70, 1.50 0.77, 1.51 0.65, 1.60 0.91, 1.21 0.47, 2.33 0.79, 1.53 /	0.63 / 0.13 0.518 1.00 / / 0.89 0.65 0.94 0.49 0.92 0.56 /	I2 52%, P = 0.06 / I2 45% I2 79.6% I2 0%, P = 0.48 / / I2 56%, P = 0.13 I2 0%, P = 0.57 I2 0%, P = 0.68 I2 0%, P = 0.85 / I2 38%, P = 0.21 /
Overall Survival	(20)° (5-year) (21)° (22)° (5-year) (24)° (26)° (27)° (28)° (29)* (1 year) (29)* (3 year) (29)* (5 year) (29)° (1 year) (29)° (3 year) (29)° (5 year) (30)	0.88 1.18 1.33 0.844 1.15 / / 1.01 1.07 1.04 1.01 0.95 1.01 0.87	0.49, 1.58 0.84, 1.65 0.86, 2.07 0.412, 1.730 0.53, 2.50 / / 0.96, 1.06 0.86, 1.34 0.84, 1.28 0.98, 1.05 0.82, 1.10 0.82, 1.25 0.77, 0.99	0.68 0.33 0.20 0.644 0.73 / / 0.48 0.53 0.75 0.48 0.49 0.92 0.03	I2 72%, P = 0.68 I2 0%, P = 0.47 I2 41% I2 80.6% I2 0%, P = 0.94 / / I2 0%, P = 0.75 I2 61%, P = 0.08 I2 0%, P = 0.38 I2 0%, P = 0.75 I2 0%, P = 0.72 I2 37%, P = 0.19 I2 0%, P = 0.67

*Only RCT.

°Only NRCT. SMD, Standardized Mean Difference; MD, Mean Deviation; OR, Odds Ratio; RR, Relative Risk; HR, Hazard Ratio; CI, Confidence Interval.

### Blood loss

The analysis of blood loss consistently favored the minimally invasive group across all meta-analyses from non-randomized studies (20–22, 24, 26–29). In the assessment of this parameter within randomized controlled trials (RCTs), Ozair et al. (29) observed a lower estimated blood loss (EBL) in the MIS group. Although not statistically significant, this information aligns with the findings of observational studies, consistently reporting significantly reduced EBL with minimally invasive hepatectomy. This unanimity in findings underscores that laparoscopic/MIS techniques significantly reduce intraoperative blood loss, which can be crucial in minimizing the risk of complications and ensuring patient safety.

### Blood transfusion

When assessing the need for blood transfusion, eight meta-analyses from non-randomized studies (20–22, 24, 26–29) reported a lower rate in the MIS group compared to the open liver resection group. The finding suggests that patients undergoing MIS are less likely to require blood transfusions, signifying a potential advantage in blood preservation. When evaluating this parameter within randomized controlled trials (RCTs), a lower, although not statistically significant, need for transfusion was reported with minimally invasive hepatectomy (29).

### Duration of surgery

The analysis of duration of surgery across seven meta-analyses revealed no significant difference between the MIS group and the open liver resection group (20–22, 24, 26–28), suggesting that, in most cases, MIS does not significantly extend the duration of the procedure.

### Complication rate

Seven meta-analyses of non-RCTs (20–22, 24, 27–29) indicated a lower rate of perioperative complications in the MIS group, emphasizing the potential benefit of MIS in reducing post-operative complications. However, one included study (26) found no significant difference between the two groups, suggesting that the effectiveness of MIS in reducing complications may depend on specific patient characteristics or procedural factors, such as patient fitness, the presence of comorbidities, or the surgeon’s experience and used technique. Evidence from RCTs revealed a lower risk of complications with minimally invasive liver resections (29).

### Hospitalization time

The analysis of hospitalization time revealed that seven meta-analyses from non-RCTs (20–22, 26–29) detected a shorter hospital stay for patients in the MIS group. Correspondingly, data from RCTs align with these findings (29), supporting the notion that MIS promotes faster post-operative recovery and reduces hospitalization duration. However, one meta-analysis (24) found no significant difference between the two groups, indicating that other factors may influence the length of hospitalization.

### Post-operative mortality

Among the included meta-analyses, five considered post-operative mortality as an operative outcome. All five of the cited studies (20–22, 24, 27) reported no significant difference in mortality rates between the MIS and open liver resection groups.

### Surgical margins R0

Among the meta-analyses that evaluated this oncologic outcome, three reported higher rates of surgical margins R0 in the MIS group (21, 22, 28). However, one study (29) reported nearly identical rates of R0 resection between the two groups. Another study (20) indicated a slightly higher rate of R0 margins in the open liver resection (OLR) group, highlighting potential variability in outcomes. One meta-analysis (24) found a lower incidence of R1 resection in the LLR group, however, Luo at al (27). did not find any significant difference in terms of increased R1 positive margins between the two groups. Data from RCTs (29) did not detect any significant difference between the MIS and OLR groups.

### Recurrence

Regarding cancer recurrence, three meta-analyses were included in the analysis. While two of these meta-analyses (20, 21) reported a lower recurrence rate in the MIS group, the statistical significance was not reached in the latter. These findings imply a potential advantage of MIS in controlling cancer recurrence. However, a third meta-analysis (22) did not find a statistically significant difference between the two groups, indicating the need for additional research to comprehensively assess the impact of MIS on recurrence rates.

### Overall survival and disease-free survival

Data from eight meta-analyses (20–22, 24, 26–29), presented no significant difference was observed between the MIS and open liver resection groups regarding overall survival and disease-free survival. Notably, Syn et al. (30), in their meta-analysis of Individual Patient Data From Randomized Trials and Propensity-score Matched Studies, reported a consistent survival advantage favoring laparoscopic over open hepatectomy for colorectal liver metastases (CLM).

- (In **Table 4**, we assessed outcomes pertaining to minimally invasive versus open hepatectomy for CRLM, specifically when performed simultaneously with the resection of the primary tumor).

**Table 4:** Results for different outcomes in patients undergoing laparoscopic versus open simultaneous resection of CRLM and primary CRC.

Outcomes	Reference	SMD/MD/OR/ RR/HR	95% CI	P-value	Test of heterogeneity
Blood loss	(21)° (23)° (25)° (29)°	-161.32 -113.31 -155.85 -177.35	-377.28, `54.64 -189.03, -37.59 -305.64, -6.06 -273.17, -104.03	0.14 0.003 0.04 0.0003	I2 95%, P < 0.00001 I2 91.4% / I2 92%, P < 0.00001
Blood transfusion	(21)° (23)° (25)° (29)°	/ / 0.61 0.92	/ / 0.29, 1.28 0.58, 1.45	/ / 0.19 0.71	/ / I2 0%, P = 0.86 I2 0%, P = 0.91
Duration of surgery	(21)° (23)° (25)° (29)°	/ 36.57 37.35 /	/ 7.80, 65.35 6.22, 80.92 /	/ 0.013 0.09 /	/ I2 82.4% I2 86%, P < 0.00001 /
Complication rate	(21)° (23)° Propensity group Non-propensity group (25)° (29)°	0.99 0.81 0.49 0.89 0.68	0.43, 2.29 0.51, 1.31 0.27, 0.88 0.56, 1.43 0.42, 1.12	0.99 0.388 0.016 0.64 0.13	I2 0%, P = 0.40 / / I2 0%, P = 0.81 I2 0%, P = 0.86
Hospitalization Time	(21)° (23)° (25)° (29)°	-3.40 -3.20 -3.16 -3.00	-4.37, -2.44 -5.06, -1.34 -4.00, -2.31 -3.82, -2.17	< 0.00001 0.001 < 0.00001 < 0.00001	I2 42%, P = 0.19 / I2 45%, P = 0.12 I2 48%, P = 0.04
Post-operative mortality	(21)° (23)° (25)° (29)°	/ / / /	/ / / /	/ / / /	/ / / /
Surgical Margins R0	(21)° (23)° (25)° (29)°	/ / / 1.02	/ / / 0.98, 1.05	/ / / 0.37	/ / / I2 34%, P = 0.17
Recurrence	(21)° (23)° (25)° (29)°	/ / / /	/ / / /	/ / / /	/ / / /
Disease Free Survival	(21)° (23)° (1 year) (23)° (3 year) (25)° (29)° (1 year) (29)° (3 year) (29)° (5 year)	/ 1.05 0.66 1.82 0.98 1.02 /	/ 0.59, 1.86 0.41, 1.08 0.70, 4.74 0.54, 1.78 0.83, 1.25 /	/ 0.86 0.097 0.22 0.94 0.85 /	/ I2 0% / I2 61%, P = 0.08 I2 53%, P = 0.14 I2 0%, P = 0.42 /
Overall Survival	(21)° (23)° (1 year) (23)° (3 year) (23)° (5 year) (25)° (29)° (1 year) (29)° (3 year) (29)° (5 year)	0.86 0.56 0.94 0.69 1.72 1.03 0.94 1.26	0.30, 2.49 0.23, 1.33 0.53, 1.65 0.29, 1.68 0.62, 4.82 0.93, 1.15 0.83, 1.07 0.59, 2.70	0.78 0.187 0.822 0.417 0.30 0.51 0.34 0.55	I2 0%, P = 0.68 I2 0% I2 0% I2 0% I2 0%, P = 0.89 I2 0%, P = 0.88 I2 0%, P = 1.00 I2 0%, P = 0.68

*Only RCT.

°Only NRCT. SMD, Standardized Mean Difference; MD, Mean Deviation; OR, Odds Ratio; RR, Relative Risk; HR, Hazard Ratio; CI, Confidence Interval.

- (In **Table 5**, we present a citation matrix that details the primary studies and meta-analyses).

**Table 5:** Citation matrix; PS, Primary Studies, MA, Meta-Analysis.

MA PS	Tian et al.	Wei et al.	Zhou et al.	Pan et al.	Luo et al.	Guo et al.	Ye et al.	Schiffman et al.	Kelly et al.	Ozair et al.	Syn et al.
Abu Hilal et al. (2010)	X	X	X		X						
Beppu et al. (2015)	X										X
Castaing et al. (2009)	X	X	X		X			X	X		
Cheung et al. (2012)	X	X	X					X		X	
Guerron et al. (2012)	X	X	X		X			X	X	X	
Inoue et al. (2013)	X	X			X					X	
Iwahashi et al. (2013)	X	X									
Kubota et al. (2014)	X										
Mala et al. (2002)	X	X	X		X					X	
RMC et al. (2012)	X										
YH et al. (2015)	X										
Qiu et al. (2013)	X	X	X					X		X	
Topal et al. (2012)	X	X	X		X			X			
Cannon et al. (2012)		X	X		X			X	X		X
Chen KY et al. (2011)		X		X		X	X			X	
Doughtie et al. (2013)		X								X	
Hu MG et al. (2012)		X		X		X	X	X		X	
Huh et al. (2012)		X		X						X	
Chen YW et al. (2019)				X			X			X	
Gorgun et al. (2017)				X			X				
Ivanecz et al. (2017)				X			X			X	
Jung KU et al. (2014)				X						X	
Lin Q et al. (2015)				X		X	X				X
Ma K et al. (2018)				X			X				X
Ratti et al. (2016)				X		X	X		X	X	
Tranchart et al. (2016)				X		X	X				X
Xu X et al. (2018)				X						X	X
Takasu et al. (2014)						X	X			X	
Nguyen et al. (2011)								X			
Cipriani et al. (2016)									X	X	X
De’Angelis et al. (2015)	X								X	X	X
Hallet et al. (2017)									X	X	
Martinez Cecilia et al. (2020)									X		X
Montalti et al. (2014)									X		
Okuno et al. (2018)									X		
Shin et al. (2019)									X	X	
Kasai et al. (2018)										X	
Fretland at al. (2018)										X	X
Aghayan at al. (2021)										X	
Robles-Campos et al. (2019)										X	X
Hirokawa et al. (2013)										X	
Qiu et al. (2014)										X	
Vavra et al. (2015)										X	
Hasegawa et al. (2015)										X	
Nachmany et al. (2015)										X	
Karagkounis et al. (2016)										X	
Lewin et al. (2016)										X	
Untereiner et al. (2016)										X	X
Zeng et al. (2016)										X	X
Efanov et al. (2020)										X	
Goumard et al. (2018)										X	
Kawakatsu et al. (2020)										X	
Ratti et al. (2018)											X
Allard et al. (2015)											X

## Discussion

Laparoscopy for liver resections has come long since it was first introduced in the 1990s (31). Nowadays, it is considered a practical option for various liver surgeries, even for cases involving colorectal cancer that has spread to the liver (CRLM). This approach has gained support from studies like case series, meta-analyses, and comparisons with traditional open surgery (32).

There is a solid consensus in the medical community that laparoscopic hepatic resection is safe, feasible, and offers advantages compared to open procedures. However, using laparoscopic techniques for liver surgery is quite complicated. Surgeons need extensive training to master the skills required. The liver’s complex anatomy demands a deep understanding of its structure and the use of tools like intraoperative ultrasound to enable enhanced identification and characterization of tumors, directing intraoperative procedures (33, 34). Moreover, applying laparoscopic techniques becomes even more intricate in oncologic surgery. Adherence to radical resection criteria is paramount, necessitating a meticulous and nuanced approach. The surgeon must balance the intricacies of minimally invasive surgery (MIS) with the imperative to achieve the necessary oncological outcomes while preserving the patient’s overall well-being.

The results of this review’s comprehensive analysis shed light on the comparative outcomes of MIS, particularly laparoscopic liver resection (LLR) versus open liver resection (OLR) in the context of colorectal cancer liver metastasis (CRLM). Findings provide valuable insights into the advantages and limitations of these surgical approaches, contributing to the ongoing dialogue surrounding the optimal treatment strategy for this challenging condition.

A pivotal investigation in this domain is the OSLO-COMET Randomized Controlled Trial (RCT) (35), which, notably, was not incorporated into the included meta-analysis. Nevertheless, it is worth highlighting that our findings exhibit striking congruence with the OSLO-COMET study, particularly in the context of reduced postoperative complications observed in the LLR group when compared to OLR. In addition to the OSLO-COMET trial, another RCT, conducted by the same research group 3-years later, reported comparable survival outcomes between the LLR and OLR groups (36). Importantly, this review yields findings that are consonant with this data, further reinforcing the assertion that there may be no substantial survival advantage associated with either surgical approach.

Another noteworthy randomized controlled trial to discuss is the LapOpHuva, which reported no significant differences in short-term outcomes, including surgical duration, blood loss, transfusion requirements, or mortality. Moreover, it demonstrated similar oncological outcomes to OLR (37). These results are consistent with the findings of this umbrella review, further corroborating the notion that LLR can yield comparable outcomes to OLR across various dimensions of surgical and oncological evaluation.

A key observation from the analysis is that MIS does not significantly prolong the duration of surgery in most cases compared to OLR. The result dispels concerns about excessively prolonged surgeries associated with laparoscopic liver resections and highlights that careful patient selection and surgical planning are pivotal factors in optimizing operative durations. Furthermore, LLR is associated with a lower rate of blood transfusion and significantly reduced intraoperative blood loss. These outcomes underscore the potential advantages of MIS in terms of minimizing the need for blood products and preserving hemostasis. The benefits of reduced blood loss extend beyond transfusion-related concerns, as they may also contribute to decreased post-operative complications and expedited recovery. While the majority of meta-analyses, incorporating data from both non-randomized controlled trials (non-RCTs) and RCTs, indicated a favorable trend toward lower complication rates with minimally invasive surgery (MIS), it is noteworthy that a singular study did not detect a significant difference between MIS and open liver resection. These findings underscore the intricacies involved in evaluating complication rates, emphasizing the impact of different factors such as patient comorbidities and the specific surgical techniques employed.

Nonetheless, the potential reduction in perioperative complications associated with MIS remains a compelling aspect, potentially improving the overall safety profile of these procedures. The analysis consistently showed that MIS is associated with a shorter hospitalization time. The finding aligns with the concept of minimally invasive surgery promoting faster post-operative recovery and shorter lengths of stay, which can lead to substantial cost savings and improved patient satisfaction.

The analysis of surgical margins (R0) in the context of liver resections for colorectal cancer metastasis presents a complex and multifaceted picture. While some meta-analyses suggest a potential advantage in achieving R0 resections with LLR, variations in outcomes, as highlighted by individual studies and RCTs, underscore the need for cautious interpretation. The choice between LLR and open techniques should be tailored to the specific characteristics of the tumor and the nuances of the anatomical context, recognizing the intricacies involved in achieving optimal oncologic outcomes.

Concerning cancer recurrence, although two meta-analyses reported a reduced recurrence rate in the MIS group, it is crucial to note that statistical significance was not observed in one of these studies. This outcome underscores the importance of ongoing research to delineate the impact of MIS on recurrence rates and to elucidate the patient subgroups that may benefit most from this approach.

The disparity in findings regarding survival outcomes is likely influenced by variations in study populations, methodologies, and the inclusion of different types of studies. This highlights the intricacies involved in comparing outcomes in surgical interventions and underscores the importance of considering diverse factors when interpreting results from meta-analyses. The meta-analysis conducted by Syn et al. (30), which integrates individual patient data and propensity-score matched studies, offers a more detailed and patient-specific perspective. This approach has the potential to capture nuanced differences that broader analyses may overlook. The identification of potentially improved survival among patients undergoing laparoscopic liver resections introduces a new perspective that warrants further investigation.

Presently, MIS is embarking on a new era with the integration of robotic technology into clinical practice. Although it initially made strides in urologic procedures, robotic applications have now branched out into various surgical domains. Among these, it has notably risen to prominence and seen extensive use in the field of general surgery. The hallmark features of robotic surgery include high-definition 3D magnified vision, endo-wristed movements, precision, and surgical finesse. These characteristics have effectively surmounted some of the technical constraints associated with laparoscopic surgery. As a result, they have garnered significant recognition, firmly establishing robot-assisted liver surgery as a universally accepted approach for the management of a wide range of hepatic conditions. In 2010, Giulianotti et al. published a pioneering series comprising a total of 70 cases of robotic hepatectomies. This initial experience provided compelling evidence of the safety of the robotic approach in liver resections, as demonstrated by low rates of conversion, minimal bleeding, and postoperative complications (38). In 2018, a significant milestone was achieved when the Asian group led by Rong Liu recorded the first consensus regarding robotic hepatectomies (39). Their findings yielded strong recommendations for the safety and efficacy of robotic procedures when compared to both open (2C) and laparoscopic (2D) approaches. Furthermore, the comparison with open hepatectomies (OD) for malignancies garnered a 2D recommendation. Notably, even the indication for living-donor robotic hepatectomy received a 2D recommendation, underscoring the growing acceptance and endorsement of this advanced surgical modality.

Today the majority of the studies found in the literature consider robotic liver surgery a safe approach and effective approach to liver malignancies as for the laparoscopic approach (40–43). There is wide acceptance among surgeons of the use of robotic surgery in complex cases like in cirrhotic patients or delicate procedures requiring, for example, micro-suturing, vascular resections (44), or bilio-enteric anastomosis (43, 45). However, it’s important to note that standardization of many of the techniques within this approach has not yet been fully realized and no research has provided conclusive guidelines for when to recommend or discourage robotic surgery due to the absence of randomized control trials (46).

The study may face limitations regarding the availability and quality of the primary research studies included in the umbrella review. Heterogeneity among the included studies could affect the overall conclusions. However, rigorous inclusion criteria were applied to ensure the reliability of the selected studies.

## Conclusion

In conclusion, this analysis indicates that laparoscopic liver resections exhibit notable advantages over open liver resections. The observed reductions in blood loss, decreased transfusion requirements, and shorter hospitalization times suggest that adopting laparoscopic approaches could contribute to more efficient and patient-friendly postoperative experiences. Moreover, the lower complication rates associated with laparoscopy indicate a potential enhancement in the overall safety profile of these procedures. These practical implications are particularly relevant in the context of personalized treatment strategies, where consideration of patient-specific factors and tumor characteristics plays a crucial role in decision-making. To improve our understanding of laparoscopic liver resections’ oncological efficacy and long-term impact, there is a compelling need for additional high-quality randomized controlled trials (RCTs) and multicentric observational studies. These studies will not only contribute crucial insights into the intervention’s effectiveness but also address the complexities inherent in comparing outcomes across diverse patient populations.

## Data availability statement

The original contributions presented in the study are included in the article/**Supplementary Materials**. Further inquiries can be directed to the corresponding author.

## Author contributions

FP: Methodology, Writing – original draft. MDP: Writing – original draft. AMar: Conceptualization, Methodology, Supervision, Writing – original draft, Writing – review & editing. LT: Writing – original draft. FT: Writing – original draft. LC: Writing – original draft. MC: Methodology, Writing – original draft. AMat: Writing – original draft. GB: Writing – review & editing. GS: Writing – review & editing. SA: Supervision, Writing – review & editing. FG: Conceptualization, Methodology, Supervision, Writing – review & editing.

## References

[1] Taillieu E De Meyere C Nuytens F Verslype C D’Hondt M . Laparoscopic liver resection for colorectal liver metastases — short- and long-term outcomes: A systematic review. World J Gastrointest Oncol. (2021) 13:732–57. doi: 10.4251/wjgo.v13.i7.732 PMC8299931 34322201

[2] Troisi R Montalti R Smeets P Van Huysse J Van Vlierberghe H Colle I . The value of laparoscopic liver surgery for solid benign hepatic tumors. Surg Endosc. (2008) 22:38–44. doi: 10.1007/s00464-007-9527-y 17705077

[3] Köckerling F . Robotic vs. Standard laparoscopic technique – what is better? Front Surg. (2014) 1:15. doi: 10.3389/fsurg.2014.00015 25593939 PMC4286948

[4] Nguyen KT Gamblin TC Geller DA . World review of laparoscopic liver resection—2,804 patients. Ann Surg. (2009) 250:831. doi: 10.1097/SLA.0b013e3181b0c4df 19801936

[5] Lo WM Tohme ST Geller DA . Recent advances in minimally invasive liver resection for colorectal cancer liver metastases—A review. Cancers. (2022) 15:142. doi: 10.3390/cancers15010142 36612137 PMC9817853

[6] Reich H McGlynn F DeCaprio J Budin R . Laparoscopic excision of benign liver lesions. Obstet Gynecol. (1991) 78:956–8. 1833688

[7] Katkhouda N Fabiani P Benizri E Mouiel J . Laser resection of a liver hydatid cyst under videolaparoscopy. Br J Surg. (1992) 79:560–1. doi: 10.1002/bjs.1800790628 1535261

[8] Gagner M Rheault M Dubuc J . Laparoscopic partial hepatectomy for liver tumor. Surg Endosc. (1992) 6:97–8.

[9] Azagra JS Goergen M Gilbart E Jacobs D . Laparoscopic anatomical (hepatic) left lateral segmentectomy—technical aspects. Surg Endosc. (1996) 10:758–61. doi: 10.1007/BF00193052 8662435

[10] Kaneko H Takagi S Shiba T . Laparoscopic partial hepatectomy and left lateral segmentectomy: Technique and results of a clinical series. Surgery. (1996) 120:468–75. doi: 10.1016/S0039-6060(96)80065-1 8784399

[11] Buell JF Cherqui D Geller DA O’Rourke N Iannitti D Dagher I . The international position on laparoscopic liver surgery: the louisville statement, 2008. Ann Surg. (2009) 250:825. doi: 10.1097/SLA.0b013e3181b3b2d8 19916210

[12] Wakabayashi G Cherqui D Geller DA Buell JF Kaneko H Han HS . Recommendations for laparoscopic liver resection: a report from the second international consensus conference held in Morioka. Ann Surg. (2015) 261:619–29. 10.1097/SLA.0000000000001184 25742461

[13] Cho JY Han HS Kaneko H Wakabayashi G Okajima H Uemoto S . Survey results of the expert meeting on laparoscopic living donor hepatectomy and literature review. Dig Surg. (2017) 35:289–93. doi: 10.1159/000479243 29032378

[14] Abu Hilal M Al E Clavien PA . The southampton consensus guidelines for laparoscopic liver surgery: from indication to implementation. Ann Surg. (2018) 268:11–8. doi: 10.1097SLA.0000000000002524 10.1097/SLA.0000000000002524 29064908

[15] Martinino A Pereira JPS Spoletini G Treglia G Agnes S Giovinazzo F . The use of the T-tube in biliary tract reconstruction during orthotopic liver transplantation: An umbrella review. Transplant Rev Orlando Fla. (2022) 36:100711. doi: 10.1016/j.trre.2022.100711 35843181

[16] Fusar-Poli P Radua J . Ten simple rules for conducting umbrella reviews. BMJ Ment Health. (2018) 21:95–100. doi: 10.1136/ebmental-2018-300014 PMC10270421 30006442

[17] Shea BJ Reeves BC Wells G Thuku M Hamel C Moran J . AMSTAR 2: a critical appraisal tool for systematic reviews that include randomised or non-randomised studies of healthcare interventions, or both. BMJ. (2017) 358:j4008. doi: 10.1136/bmj.j4008 28935701 PMC5833365

[18] Eriksen MB Frandsen TF . The impact of patient, intervention, comparison, outcome (PICO) as a search strategy tool on literature search quality: a systematic review. J Med Libr Assoc JMLA. (2018) 106:420–31. doi: 10.5195/jmla.2018.345 PMC6148624 30271283

[19] Page MJ McKenzie JE Bossuyt PM Boutron I Hoffmann TC Mulrow CD . The PRISMA 2020 statement: an updated guideline for reporting systematic reviews. Syst Rev. (2021) 10:89. doi: 10.1186/s13643-021-01626-4 33781348 PMC8008539

[20] qiang TZ fang S yong LZ chao W xin WL He J . Meta-analysis of laparoscopic versus open liver resection for colorectal liver metastases. Oncotarget. (2016) 7:84544–55. doi: 10.18632/oncotarget.v7i51 PMC5356680 27811369

[21] Wei M He Y Wang J Chen N Zhou Z Wang Z . Laparoscopic versus Open Hepatectomy with or without Synchronous Colectomy for Colorectal Liver Metastasis: A Meta-Analysis. PloS One. (2014) 9:e87461. doi: 10.1371/journal.pone.0087461 24489916 PMC3906170

[22] Zhou Y Xiao Y Wu L Li B Li H . Laparoscopic liver resection as a safe and efficacious alternative to open resection for colorectal liver metastasis: a meta-analysis. BMC Surg. (2013) 13:44. doi: 10.1186/1471-2482-13-44 24083369 PMC3849970

[23] Pan L Tong C Fu S Fang J Gu Q Wang S . Laparoscopic procedure is associated with lower morbidity for simultaneous resection of colorectal cancer and liver metastases: an updated meta-analysis. World J Surg Oncol. (2020) 18:251. doi: 10.1186/s12957-020-02018-z 32958079 PMC7507629

[24] Luo LX Yu ZY Bai YN . Laparoscopic hepatectomy for liver metastases from colorectal cancer: A meta-analysis. J Laparoendosc Adv Surg Tech. (2014) 24:213–22. doi: 10.1089/lap.2013.0399 24571350

[25] Guo Y Gao Y Chen G Li C Dong G . Minimally invasive versus open simultaneous resections of colorectal cancer and synchronous liver metastases: A meta-analysis. Am Surg. (2018) 84:192–200. doi: 10.1177/000313481808400224 29580345

[26] Ye SP Qiu H Liao SJ Ai JH Shi J . Mini-invasive vs open resection of colorectal cancer and liver metastases: A meta-analysis. World J Gastroenterol. (2019) 25:2819–32. doi: 10.3748/wjg.v25.i22.2819 PMC6580357 31236004

[27] Schiffman SC Kim KH Tsung A Marsh JW Geller DA . Laparoscopic versus open liver resection for metastatic colorectal cancer: A metaanalysis of 610 patients. Surgery. (2015) 157:211–22. doi: 10.1016/j.surg.2014.08.036 25282529

[28] Kelly ME Fahy M Bolger JC Boland PA Neary C McEntee GP . Open versus laparoscopic liver resection of colorectal metastases: a meta-analysis of matched patient populations. Ir J Med Sci. (2022) 191:1531–8. doi: 10.1007/s11845-021-02780-3 34535883

[29] Ozair A Collings A Adams AM Dirks R Kushner BS Sucandy I . Minimally invasive versus open hepatectomy for the resection of colorectal liver metastases: a systematic review and meta-analysis. Surg Endosc. (2022) 36:7915–37. doi: 10.1007/s00464-022-09612-0 36138246

[30] Syn NL Kabir T Koh YX Tan HL Wang LZ Chin BZ . Survival advantage of laparoscopic versus open resection for colorectal liver metastases: A meta-analysis of individual patient data from randomized trials and propensity-score matched studies. Ann Surg. (2020) 272:253–65. doi: 10.1097/SLA.0000000000003672 32675538

[31] Morise Z Wakabayashi G . First quarter century of laparoscopic liver resection. World J Gastroenterol. (2017) 23:3581–8. doi: 10.3748/wjg.v23.i20.3581 PMC5449415 28611511

[32] Coelho FF Kruger JAP Fonseca GM Araújo RLC Jeismann VB Perini MV . Laparoscopic liver resection: Experience based guidelines. World J Gastrointest Surg. (2016) 8:5–26. doi: 10.4240/wjgs.v8.i1.5 26843910 PMC4724587

[33] Nanashima A Tobinaga S Abo T Kunizaki M Takeshita H Hidaka S . Usefulness of sonazoid–ultrasonography during hepatectomy in patients with liver tumors: A preliminary study. J Surg Oncol. (2011) 103:152–7. doi: 10.1002/jso.21782 21259249

[34] Zacherl J Scheuba C Imhof M Zacherl M Längle F Pokieser P . Current value of intraoperative sonography during surgery for hepatic neoplasms. World J Surg. (2002) 26:550–4. doi: 10.1007/s00268-001-0266-2 12098044

[35] Fretland ÅA Dagenborg VJ Bjørnelv GMW Kazaryan AM Kristiansen R Fagerland MW . Laparoscopic versus open resection for colorectal liver metastases: the OSLO-COMET randomized controlled trial. Ann Surg. (2018) 267:199. doi: 10.1097/SLA.0000000000002353 28657937

[36] Aghayan DL Kazaryan AM Dagenborg VJ Røsok BI Fagerland MW Waaler Bjørnelv GM . Long-term oncologic outcomes after laparoscopic versus open resection for colorectal liver metastases. Ann Intern Med. (2021) 174:175–82. doi: 10.7326/M20-4011 33197213

[37] Robles-Campos R Lopez-Lopez V Brusadin R Lopez-Conesa A Gil-Vazquez PJ Navarro-Barrios Á . Open versus minimally invasive liver surgery for colorectal liver metastases (LapOpHuva): a prospective randomized controlled trial. Surg Endosc. (2019) 33:3926–36. doi: 10.1007/s00464-019-06679-0 30701365

[38] Giulianotti PC Coratti A Sbrana F Addeo P Bianco FM Buchs NC . Robotic liver surgery: Results for 70 resections. Surgery. (2011) 149:29–39. doi: 10.1016/j.surg.2010.04.002 20570305

[39] Liu R Wakabayashi G Kim HJ Choi GH Yiengpruksawan A Fong Y . International consensus statement on robotic hepatectomy surgery in 2018. World J Gastroenterol. (2019) 25:1432–44. doi: 10.3748/wjg.v25.i12.1432 PMC6441912 30948907

[40] Fahrner R Rauchfuß F Bauschke A Kissler H Settmacher U Zanow J . Robotic hepatic surgery in Malignancy: review of the current literature. J Robot Surg. (2019) 13:533–8. doi: 10.1007/s11701-019-00939-w 30895519

[41] Tsilimigras DI Moris D Vagios S Merath K Pawlik TM . Safety and oncologic outcomes of robotic liver resections: A systematic review. J Surg Oncol. (2018) 117:1517–30. doi: 10.1002/jso.25018 29473968

[42] Qiu J Chen S Chengyou D . A systematic review of robotic-assisted liver resection and meta-analysis of robotic versus laparoscopic hepatectomy for hepatic neoplasms. Surg Endosc. (2016) 30:862–75. doi: 10.1007/s00464-015-4306-7 26092026

[43] Ocuin LM Tsung A . Robotic liver resection for Malignancy: Current status, oncologic outcomes, comparison to laparoscopy, and future applications. J Surg Oncol. (2015) 112:295–301. doi: 10.1002/jso.23901 26119652

[44] Magistri P Pang NQ Guidetti C Caracciolo D Odorizzi R Catellani B . Robotic approach for perihilar cholangiocarcinoma: from Bismuth 1 to vascular resection. Eur J Surg Oncol. (2023) 49:107002. doi: 10.1016/j.ejso.2023.107002 37599146

[45] Peters BS Armijo PR Krause C Choudhury SA Oleynikov D . Review of emerging surgical robotic technology. Surg Endosc. (2018) 32:1636–55. doi: 10.1007/s00464-018-6079-2 29442240

[46] Robotic liver surgery: literature review and current evidence. Mini-Invasive Surg. (2020) 4:null–l. doi: 10.20517/2574-1225.2020.90

